# Postoperative ecchymoma of eyelid after botulinum toxin injection for hemifacial spasm: a case report

**DOI:** 10.3389/fneur.2023.1171303

**Published:** 2023-07-20

**Authors:** Xinyu Hu, Kexin Guo, Jingwen Li, Xinyi Wang, Hanshu Liu, Qinwei Yu, Guiying Kuang, Gang Li, Jinsha Huang, Hongge Li, Zhicheng Lin, Nian Xiong

**Affiliations:** ^1^Department of Neurology, Tongji Medical College, Union Hospital, Huazhong University of Science and Technology, Wuhan, China; ^2^Department of Cardiology, Wuhan Red Cross Hospital, Wuhan, China; ^3^Department of Neurology, Wuhan Red Cross Hospital, Wuhan, China; ^4^Laboratory of Psychiatric Neurogenomics, McLean Hospital, Harvard Medical School, Belmont, MA, United States

**Keywords:** postoperative ecchymoma, eyelid, botulinum toxin injection, hemifacial spasm, case report

## Abstract

Hemifacial spasm (HFS) is a rare movement disorder characterized by involuntary muscle contractions on one side of the face. Compared to the high therapeutic effect, adverse effects of botulinum toxin treatment for HFS occurred rarely. However, managing HFS patients who are also taking antithrombotic drugs poses a challenge. Here, we present a case of postoperative ecchymoma of the eyelid following a botulinum toxin injection in a patient receiving daily vinpocetine and aspirin antiplatelet therapy. This case highlights the importance of considering the potential risks and formulating a treatment plan that maximizes benefit while minimizing complications in HFS patients undergoing botulinum toxin injections and taking antithrombotic medications. To the best of our knowledge, this is the first reported case of postoperative ecchymoma of the eyelid following a botulinum toxin injection. Further research and additional case reports are needed to better understand the management strategies for this patient population.

## Introduction

Hemifacial spasm (HFS) is a rare movement disorder characterized by involuntary muscle contractions on one side of the face (1). Primary HFS has proven to be a neurovascular compression syndrome of VII cranial nerves by aberrant arteries. The mean annual incidence is 0.81 per 100,000 women and 0.74 per 100,000 men (2). And the mean age at disease onset is 55 years. Usually, HFS initially involves the orbicularis oculi muscle, followed by gradually spreading to other parts of the face, which causes significant negative impact on the quality of life in HFS patients (3). Without proper therapy, the symptoms may last for lifetime, and the spasms progress gradually in terms of intensity and frequency (2). Pharmacological treatments, including carbamazepine, clonazepam, baclofen as well as other anticonvulsive drugs such as gabapentin, can be beneficial in some patients, but such treatments can be limited in severe and disabling cases by serious side effects (4). Botulinum toxin (BTX), produced by *Clostridium botulinum*, is a neurotoxin that paralyses muscles by irreversibly blocking the cholinergic signal transmission at the presynaptic nerve endings (5). For a long time, BTX has only been used in cosmetic dermatology. Since the 1980s, botulinum toxin type A (BTXA) has been used to treat HFS and provide symptom relief and improved quality of life in about 85 to 95% of the cases (6). Recently, botulinum toxin A (BTXA) has been introduced as a safe and effective therapeutic option for HFS (7). Most patients achieve moderate or marked relief by accepting BTXA therapy, although the injection must be repeated every 3 to 6 months. The intramuscular injections may cause various adverse effects including mild facial paresis, diplopia, allergy, pain, lid ptosis, brow ptosis, or hematomas. However, the available evidence suggests that hematoma at injection sites occur in less than 3% of BTXA injections (8) and no serious systemic side effects of the therapy have been reported yet (9).

In this case report, we describe an elder patient who was treated with aspirin and vinpocetine, showing ecchymoma once BTX injected. We aim to raise the question whether the antiplatelet or anticoagulant drugs should be discontinued before the BTXA therapy.

### Case report

A 60-year-old man, previously diagnosed with posterior circulation ischemia, had a 7-year history of right-sided HFS. Initially he had intermittent involuntary contractions of the right eyelid, which resulted in forced closure of the eye, and the spasms gradually spread to muscles of the lower part of the face. Ultimately, these muscles on the right side were affected nearly all the time. The distinct spasm of the orbicularis oculi muscle impaired his homolateral vision, which severely impacted his ability to read. The severity of HFS was assessed by the standard of Shorr et al. (10). Grade 0: no spasm; gradeI: increased frequency of blink caused by external stimulus; gradeII: mild spasm, slight tremor of eyelid and no dysfunction; grade III: obvious spasm and mild dysfunction; gradeIV: severe spasm and severe spasm and dysfunction (unable to read and drive, etc.). In this case, prior to treatment, the severity was assessed at graded IV. Moreover, he used aspirin 100 mg daily to prevent ischemic stroke. He was referred to the Department of Neurology, Union Hospital, Tongji Medical College, Huazhong University of Science and Technology for the treatment of both posterior circulation ischemia and HFS. A computed tomography scan of the brain and angiography obtained normal results. Routine laboratory tests, including blood test, urine test, electrolytes, liver-function and renal-function tests, and erythrocyte sedimentation rate measurements all showed normal results, apart from mildly elevated total cholesterol and low density lipoprotein levels. Coagulation function test parameters were also negative.

**Figure 1:**
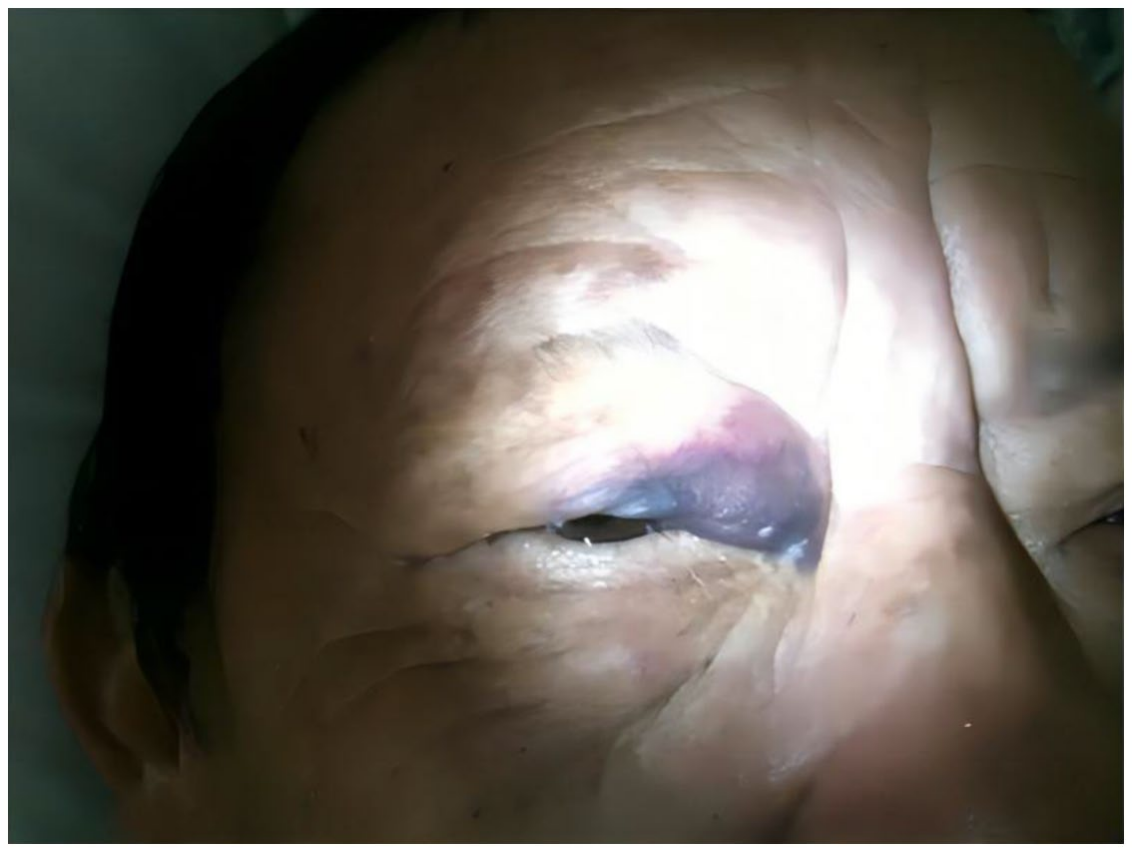
The extensive eyelid ecchymoma of the patient 12 hours after the BTXA injection.

Based on the clinical picture, the patient was being treated continuously with aspirin 100 mg and vinpocetine 30 mg daily. Furthermore, the patient was willing to receive the BTXA injection to relieve the spasm. BTXA (Botox, 100 U of clostridium botulinum type A neurotoxin complex) was acquired from Allergan Inc., the United States. BTX-A was dissolved in 2 mL of saline (0.9%) to 50 U/mL, as recommended by the manufacturer. 1 mL syringe with a 26-gauge needle was used for the injection procedure. On the right side, the dosage of BTXA per injection site was 2.5 to 5 units, including orbicularis oculi (5 points, 5 units per points), corrugator supercilii (1 point, 5 units), zygomaticus (1 point, 2.5 units) orbicularis oris (3 points, 5 units per points) and the muscle mentalis (1 point, 2.5 units) (10). To keep the balance, a half of the BTXA dosage was injected to a variety of muscles on the left side. A total of 50 units of BTXA were administered. Considering the patient’s history of antiplatelet medication, we conducted pre-injection aspiration by gently withdrawing the syringe plunger to check for the presence of blood. Additionally, we administered the BTXA injection slowly and with precise control to minimize tissue trauma and the potential risk of bleeding. We closely monitored the injection site for any signs of bleeding or hematoma formation. However, the patient developed an ipsilateral periocular swelling and eyelid hematoma the following morning, approximately 12 hours after receiving the injection (Figure 1; Supplementary Video S1). The patient was referred to an ophthalmologist for counseling. The periorbital swelling and eyelid ecchymoma were absorbed in 7 days after injection gradually (Supplementary Video S2) and disappeared 14 days later. Meanwhile, we observed mild eyelid muscle tremors without any functional impairments such as whistling, blowing, frowning, or chewing (Supplementary Video S2). The Shorr scale assessment decreased to gradeII. Moreover, the severity of spasm decreased to grade 0 after BTX treatment.

Based on our follow-up, complete remission was observed for a duration of 4 months, with symptoms gradually returning to the pre-injection level after 2 months. Notably, to prevent hematoma formation, we implemented specific techniques, including careful aspiration prior to injection and a slow and controlled injection process. Additionally, we opted for smaller insulin syringe needles, specifically a 30-gauge size, to minimize tissue trauma. Post-injection, we applied ice packs to the injection site and surrounding area to facilitate cold therapy and mitigate the risk of hematoma formation. Treatment was then repeated with the same dosage (50 units) injected in the same muscles, resulting in a similar marked improvement. Now the patient is receiving the injection every 6 months regularly and reaches satisfied remission without eyelid ecchymoma.

## Discussion

The ecchymoma of eyelid is a relatively rare and late complication after the botulinum toxin therapy for HFS. There are few reports about this issue at home and abroad. In this case, the patient developed a postoperative hematoma characterized by tardive ecchymoma, which refers to a time delay between the initial occurrence and its manifestation. The onset of ipsilateral periocular swelling and eyelid hematoma was observed the morning after receiving the botulinum toxin injection, which occurred 12 hours prior. While the majority of tardive hematomas in cosmetic procedures or facial surgeries typically manifest within 24 h to several days after the procedure, the occurrence of a hematoma 12 h after the injection may not strictly align with the clinical definition of a delayed hematoma (11, 12). Therefore, we have chosen to categorize this hematoma as a “postoperative hematoma” rather than a “tardive hematoma”.

To our knowledge, this is the first report of postoperative ecchymoma of eyelid after botulinum toxin injection. Nevertheless, patients are recommended to discontinue nonsteroidal anti-inflammatory agents such as aspirin and also tocopherol or even gingko biloba, 10 days before BTXA injections (13). In this case, the patient presented a marked ecchymoma, which may attribute to: (1) The elders usually have loose eyelid tissue and periorbital tissue which blood can easily permeate into; (2) The patient was being treated with aspirin and vinpocetine which may have increased the risk of ecchymomsis complications; and (3) Some delayed immune rejections related to the neutralizing antibodies *in vivo*. Specifically, the immune response triggered by BTXA injection can lead to the production of neutralizing antibodies, which can interfere with blood clotting mechanisms (14). These antibodies may bind to proteins involved in clotting, such as coagulation factors and platelets, impairing platelet aggregation and inhibiting coagulation factor activity. In addtion, they can induce inflammation and damage to blood vessel walls, increasing the risk of bleeding. Although the development of neutralizing antibodies in response to botulinum toxin injections is uncommon, long-term and repetitive treatment may increase this likelihood (15).

Owing to demographic characteristic changes with an aging population, the number of patients with antiplatelet or anticoagulant drugs is predictable to rise. Additionally, daily aspirin therapy is recommended by key guideline agencies for the prevention of cardiovascular and cerebrovascular events in the elderly (16). At the same time, the aged people composed a significant part of the patients who need BTX therapy (17). The elderly are more prone to bleeding in this case to some extent within the injections. In addition, the interruption of antiplatelet therapy may increase the risk of cerebral infarction or myocardial infarction leading to life-threatening, disabling, and costly consequences.

The dilemma obviously exists in patients who are receiving antiplatelet or anticoagulant therapy and still require surgery. In order to help clinicians make a decision, the American College of Chest Physicians has made cautious suggestions after considering the risk of cardiovascular events and the risk of thrombosis after the operation (18). This provides us with ideas to solve the problem. Because of the severe consequences of cardiovascular and cerebrovascular events we should evaluate the risk of discontinuing the antiplatelet therapy. For those high-risk patients, we should analyze the situation, and take into consideration the suffering ecchymoma and the risk of infarction, then formulate a treatment course to ensure maximum benefit. As for the patients who have taken anticoagulants for a long time, we are required to avoid food of high vitamin K and check coagulation function regularly. The most important thing is that the dose should be adjusted to prevent over-coagulation, if anticoagulants are used in combination with other drugs. Whether we choose the injection with discontinuing or continuing antiplatelet therapy, we should be informed of the potential risk to the patients.

Besides, efficient preparation and preventive measures are crucial in minimizing the occurrence of ecchymoma. Efficient preparation and preventive measures are crucial in minimizing the occurrence of hematoma. During injections, meticulous aspiration, slow and controlled injection techniques are important measures. Immediate pressing at the injection site after each injection have been identified as effective preventive measures against hematoma. Previous studies have also recommended the use of smaller needle sizes of appropriate length to reduce bleeding complications associated with BTX administration (19). Additionally, ultrasound guidance has shown superior therapeutic efficiency compared to relying solely on anatomical palpation (20). These measures collectively contribute to reducing the risk of hematoma formation. However, in cases where hematoma occurs, for instant application of cold compresses to the injection site can help alleviate swelling and reduce inflammatory responses (21). It is important to avoid massaging or manipulating the injection site to prevent further irritation and spreading of the hematoma. Additionally, maintaining a slightly elevated head position can reduce blood flow to the injection area and alleviate the severity of the hematoma. Close observation and monitoring of the hematoma’s changes and associated symptoms, such as swelling degree, color changes, pain, or discomfort, are necessary.

Since few literatures are available to help us make a decision, the problem that BTXA injection with discontinuing antiplatelet therapy or not should attract more attention to make a marked standard. We recommend giving due consideration to the occurrence of tardive ecchymoma and the risk of infarction by devising a treatment plan that maximizes its benefits. Furthermore, future research should aim to explore additional strategies for the prevention and management of postoperative ecchymoma.

## Data availability statement

The original contributions presented in the study are included in the article/Supplementary material, further inquiries can be directed to the corresponding author.

## Ethics statement

Written informed consent was obtained from the individual(s) for the publication of any potentially identifiable images or data included in this article.

## Author contributions

KG, JH, GL, HoL, and NX contributed to the conception and design. KG, JL, XW, HaL, QY, GK, and NX took care of collecting the clinical information. XH, KG, JL, JH and NX interoperated the images and videos. XH, KG, JL, ZL, and NX coordinated and helped to draft the manuscript. All authors contributed to the article and approved the submitted version.

## Funding

This research was funded by the National Natural Science Foundation of China (NSFC), grant number 81873782 and 82271278; 2019 Wuhan Huanghe Talents Program; 2020 Wuhan medical research project, grant number 2020020601012303; 2021 Hubei Youth Top-notch Talent Training Program and 2022 Outstanding Youth Project of Natural Science Foundation of Hubei province (all to NX).

## Conflict of interest

The authors declare that the research was conducted in the absence of any commercial or financial relationships that could be construed as a potential conflict of interest.

## Publisher’s note

All claims expressed in this article are solely those of the authors and do not necessarily represent those of their affiliated organizations, or those of the publisher, the editors and the reviewers. Any product that may be evaluated in this article, or claim that may be made by its manufacturer, is not guaranteed or endorsed by the publisher.

## Supplementary material

SUPPLEMENTARY Video S1 The eyelid ecchymoma twelve hours after the BTXA injection.

SUPPLEMENTARY Video S1 The ecchymoma seven days after the BTXA injection.
